# Supplementary material for: FXS causing missense mutations disrupt FMRP granule formation, dynamics, and function
Source: PLoS Genet. 2022 Feb 24;18(2):e1010084. doi: 10.1371/journal.pgen.1010084 (PMC8903291; doi:10.1371/journal.pgen.1010084)
Supplement: S1 Text — (DOCX) [file pgen.1010084.s001.docx]

**S1 TEXT: SUPPLEMENTAL METHODS**

**Larval NMJ immunohistochemistry and morphological analysis**

Third instar larval body wall preps for NMJ analysis were dissected in ice cold calcium-free Jan and Jan buffer (130 mM NaCl, 5 mM KCl, 36 mM sucrose, 5 mM HEPES [pH 7.3], 4 mM MgCl2, and 0.5 mM EGTA) within 30 minutes on sylgard plates. Dissection buffer was removed and preps were fixed in 4% PFA for 20 minutes, then washed 3x 5 minutes in 1X PBS (pH 7.4) and permeabilized in 1X PBS (pH 7.4) + 0.1% Triton X-100 for 10 minutes. Preps were blocked in 1X PBS with 2% BSA and 5% normal goat serum with shaking for 30 minutes. Block was removed and primary antibody (mouse anti-DLG, 1:200) diluted in block was incubated overnight at 4°C. Preps were washed 6x 5 minutes in PBS and then incubated for 1 hour with secondary antibodies diluted in block at room temperature (goat anti-mouse Alexa 488 or 567 at 1:500 & Alexa 649-conjugated anti-HRP at 1:500). Preps were washed in 1X PBS and then mounted on slides in DAPI-Fluoromount-G Clear Mounting Media. All imaging was done on an Olympus FV3000 scanning confocal microscope using 20X and 60X objectives to image the NMJ at muscles 6/7 in abdominal section 3 (N.A. 0.85 and 1.42, respectively). When shown, maximum Z projections were assembled from 0.4μm optical sections. All post-hoc image processing was done using Fiji in ImageJ2. For morphological analysis of larval NMJs, between 10-17 images were examined per experiment, in which 1s, 1b and axon terminals were manually counted at muscles 6 and 7 (m6/7) in abdominal segment 3 (A3) using the Cell Counter plugin in Fiji. To account for muscle area differences between genotypes which effects NMJ size, synaptic bouton numbers were normalized to muscle surface area (MSA). MSA was calculated by outlining both m6/7 using the freehand selection tool in ImageJ2/Fiji and recording the calculated muscle area. 1s and 1b bouton numbers were divided by the corresponding muscle area. These data were then normalized to the *C380-Gal4/+;; UAS-EGFP/+* controls. Data were collected and calculations were conducted in Excel and statistical analyses were performed in Prism.
