# Supplementary figures and images for: FXS causing missense mutations disrupt FMRP granule formation, dynamics, and function
Source: PLoS Genet. 2022 Feb 24;18(2):e1010084. doi: 10.1371/journal.pgen.1010084 (PMC8903291; doi:10.1371/journal.pgen.1010084)

Figure S1. KH domain mutants disrupt FMRP function when overexpressed in larval MNs

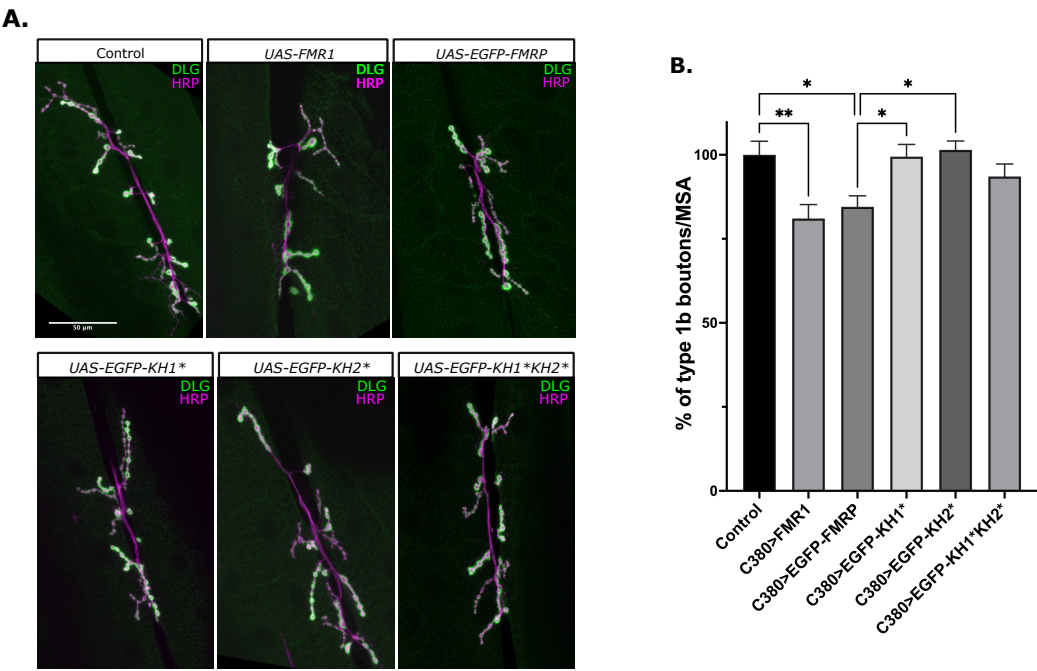

Supplement: S1 Fig — (A) Wandering third instar larval NMJs from C380-Gal4 (Control), UAS-FMR1, and UAS-EGFP:FMRP mutants were stained with antibodies targeting the postsynaptic density marker, DLG (green) and the neuronal membrane marker, HRP (magenta). Maximum Z-projections of NMJs in abdominal segment 3 innervating body wall muscles 6/7 were analyzed. Scale bar = 50μm. (B) Percentage of type 1b bouton number normalized to the area of muscles 6/7 (in μm2) was counted manually and compared with the control and EGFP:FMRP (mean ± SE; n = 10–20 NMJs; one-way ANOVA). * p<0.05, ** p<0.01. (PDF) [file pgen.1010084.s002.pdf]

Figure S2: FXS-causing mutations alter the liquid-like nature of stress granules

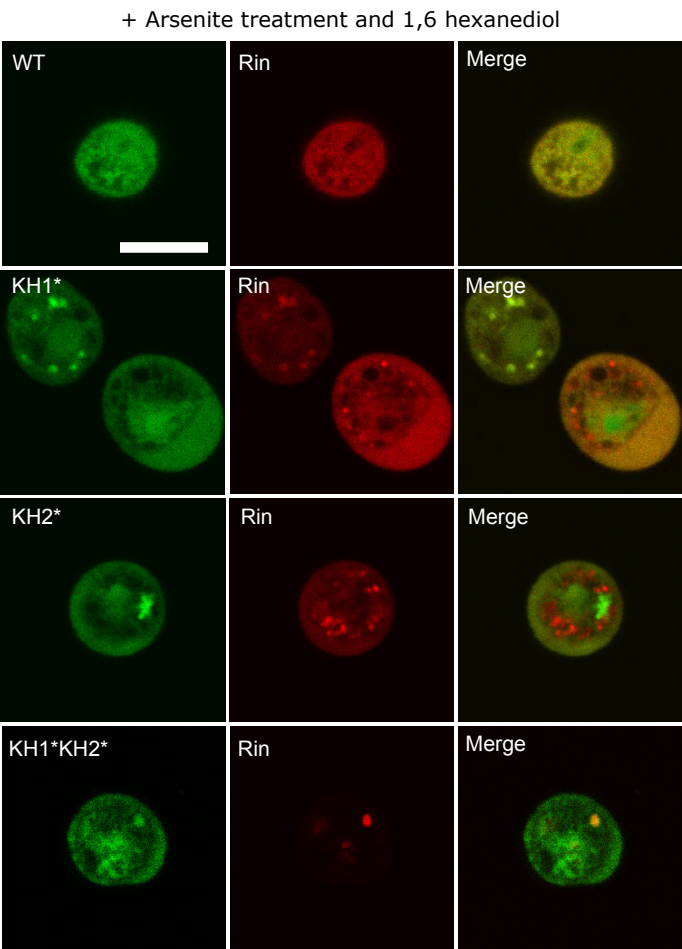

Supplement: S2 Fig — Representative images of S2R+ cells transfected with GFP-FMRP mutants (green) and Rin-mCherry (red) that have been treated with 0.5mM sodium arsenite for 45 minutes followed by treatment with 10% 1,6-HD. Scale bars = 10μm. Note that both FMRP and Rin granules fail to dissipate in KH mutant cells relative to WT-FMRP controls. (PDF) [file pgen.1010084.s003.pdf]
