# Supplementary material for: FXS causing missense mutations disrupt FMRP granule formation, dynamics, and function
Source: PLoS Genet. 2022 Feb 24;18(2):e1010084. doi: 10.1371/journal.pgen.1010084 (PMC8903291; doi:10.1371/journal.pgen.1010084)
Supplement: S1 Table — (DOCX) [file pgen.1010084.s004.docx]

**S1 Table: Fly lines**

| REAGENT or RESOURCE | SOURCE | IDENTIFIER |
| --- | --- | --- |
| *D. melanogaster: BL Canton S* | Bloomington Drosophila Stock Center | BDSC:64349 |
| *D. melanogaster: C380-Gal4* | Bloomington Drosophila Stock Center | BDSC:80580 |
| *D. melanogaster: w^1118^; UAS-FMR1* | Bloomington Drosophila Stock Center | BDSC:6931 |
| *D. melanogaster: w^1118^;; FMR1Δ50M/TM6B,Tb^+^* | Bloomington Drosophila Stock Center | BDSC:6928 |
| *D. melanogaster: w*;; FMR1^Δ113M^/TM6B,T^b+^* | Bloomington Drosophila Stock Center | BDSC:67403 |
| *D. melanogaster: C380-Gal4, cha-Gal80* | [1] |  |
| *D. melanogaster: w^1118^; wg^Sp-1^/CyO, P{w^+^mC=2xTb^1^-RFP} CyO; MKRS/TM6B, Tb^1^* | Bloomington Drosophila Stock Center | BDSC: 76359 |
| *D. melanogaster: C380-Gal4;; Sb/ TM6B,Ser* | This paper |  |
| *D. melanogaster: pUAST-attB-EGFP* | This paper |  |
| *D. melanogaster: pUAST-attB-EGFP:FMRP* | This paper |  |
| *D. melanogaster: pUAST-attB-EGFP:FMRP:KH1** | This paper |  |
| *D. melanogaster: pUAST-attB-EGFP:FMRP:KH2** | This paper |  |
| *D. melanogaster: pUAST-attB-EGFP:FMRP:KH1*KH2** | This paper |  |
| *D. melanogaster: pUAST-attB-EGFP:ΔKH* | This paper |  |
| *D. melanogaster: w+; FMR1^Δ50M^, pUAST-attB-EGFP/TM6BTb* | This paper |  |
| *D. melanogaster: w^+^; FMR1^Δ50M^, pUAST-attB-EGFP:FMRP/TM6BTb* | This paper |  |
| *D. melanogaster: w^+^; FMR1^Δ50M^, pUAST-attB-EGFP:FMRP:KH1*/TM6BTb* | This paper |  |
| *D. melanogaster: w^+^; FMR1^Δ50M^, pUAST-attB-EGFP:FMRP:KH2*/TM6BTb* | This paper |  |
| *D. melanogaster: w^+^; FMR1^Δ50M^, pUAST-attB-EGFP:FMRP:KH1*KH2*/TM6BTb* | This paper |  |
| *D. melanogaster: w^+^; FMR1^Δ50M^, pUAST-attB-EGFP:ΔKH/TM6BTb* | This paper |  |
| *D. melanogaster: C380,cha-Gal80;; TM6BTb/TM3BSb* | This paper |  |
| *D. melanogaster: C380,cha-Gal80;; TM6BTb/FMR1^Δ113M^* | This paper |  |
| *D. melanogaster: C380-Gal4;; FMR1^Δ113M^/TM6BTb* | This paper |  |

**REFERENCES:**

1. Hartwig CL, Worrell J, Levine RB, Ramaswami M, Sanyal S. Normal dendrite growth in Drosophila motor neurons requires the AP-1 transcription factor. Developmental Neurobiology. 2008;68(10):1225-42. doi: 10.1002/dneu.20655. PubMed PMID: WOS:000258259300002.
