# Supplementary material for: FXS causing missense mutations disrupt FMRP granule formation, dynamics, and function
Source: PLoS Genet. 2022 Feb 24;18(2):e1010084. doi: 10.1371/journal.pgen.1010084 (PMC8903291; doi:10.1371/journal.pgen.1010084)
Supplement: S2 Table — (DOCX) [file pgen.1010084.s005.docx]

**S2 Table: Oligonucleotides**

| REAGENT or RESOURCE | SOURCE | IDENTIFIER |
| --- | --- | --- |
| Oligonucleotides | | |
| PCR forward primer for genotyping fmr1 deletion: 5'-AAGGAAAAAAGCGGCCGCAAAGATATCGCGAAAATCCCCCCAG-3' | [1] |  |
| PCR reverse primer for genotyping fmr1 deletion 5'-CGGGATCCGTTATGCTACGTGAATAAATC-3' | [1] |  |
| Forward primer for amplifying the N-terminus of DmFMRP with a 5'-HindIII site: 5'-ACAAGCCAAGCTTTATGGAAGAT-3' | This paper |  |
| Reverse primer for amplifying the C-terminal half of DmFMRP with a 3' EcoRI site: 5'-TCTGCAGAATTCTTAGGACGTG-3' | This paper |  |
| Forward primer for amplifying the N-terminus of EGFP with a 5’ KpnI site for cloning EGFP and all EGFP:FMRP mutants into the pUAST vector: 5'-GGTACCAACATGGTGAGCAA-3' | This paper |  |
| Reverse primer for amplifying the C-terminus of EGFP with a 3' XbaI site for cloning EGFP into the pUAST vector: 5'-GTTCATCTAGACTACTTGTACAGCTCGTCCATGC-3' | This paper |  |
| Reverse primer for amplifying the C-terminal portion of FMRP without the IDR to construct ΔIDR with a 3' EcoRI site: 5'-TACGGAATTCTTACTTCTCCTGACGCAACTGTT-3' | This paper |  |
| Forward primer for amplifying the FMRP IDR with a 5' HindIII site: 5'-GTCAAAGCTTCGAGATTGATCAGCAGCTTC-3' | This paper |  |
| Forward primer for amplifying the C-terminal half of DmFMRP to construct the KH-domain deletion(ΔKH) with a 5’ BamHI site: 5'-ATGACGGATCCCTGGCGCATGTACCCTTTGT  -3' | This paper |  |
| Reverse primer for amplifying the N-terminal half of DmFMRP to construct the KH-domain deletion (ΔKH) with a 3’ BamHI site: 5’-ATGACGGATCCCTCAACGTAGTTTCCACGGC-3’ | This paper |  |
| Forward primer for SDM of the KH1 domain in dmFMRP [Gly269Glu]: 5' CAAAATCAGCGAAGAGACCGAGG -3' | This paper |  |
| Reverse primer for SDM of the KH1 domain in dmFMRP [Gly269Glu]: 5'-AATGTGCAGGACTTCTCC-3' | This paper |  |
| Forward primer for SDM of the KH2 domain in dmFMRP [Ile307Asn]: 5'-GGGCGCATTAACCAGGAGATTG-3' | This paper |  |
| Reverse primer for SDM of the KH2 domain in dmFMRP [Ile307Asn]: 5'-ATTCTTGCCAATCACCTTGC-3' | This paper |  |
| mCherry amplification forward primer with 5' HindIII site): 5'-AGTACAAGCTTATGGTGAGCAAGGGCGAGGAG-3' | This paper |  |
| mCherry amplification reverse primer with 3' BamHI site: 5'-AGTACGGATCCTTACTTGTACAGCTCGTCCATGCCG-3' | This paper |  |
| Top primer for cloning (Gly4Ser)3 linker upstream of mcherry, containing a 5' ApaI site and 3' HindIII: 5'-CGGTGGAGGAGGCTCTGGTGGAGGCGGTAGCGGAGGCGGAGGGTCGA-3' | This paper |  |
| Bottom primer for cloning (Gly4Ser)3 linker upstream of mcherry, containing ApaI and HindIII sites: 5'-AGCTTCGACCCTCCGCCTCCGCTACCGCCTCCACCAGAGCCTCCTCCACCGGGCC-3' | This paper |  |
| Rasputin RT-PCR primer with 5' KpnI site: 5'-TGACATGGTCATGGATGCGACCCA-3' | This paper |  |
| Rasputin RT-PCR primer with in-frame stop codon and 3'-EcoRI site: 5'-ATACGAATTCGCGACGTCCGTAGTTGCCA-3' | This paper |  |
| CaMKII 3'UTR Gibson assembly primer for cloning into FLuc backbone vector cut with EcoRI and XhoI: 5'-CGGAAAGTCCAAATTGTAATGGGCATTAATCAATGGAATATAAAC-3' | This paper |  |
| CaMKII 3'UTR Gibson assembly primer for cloning into FLuc backbone vector cut with EcoRI and XhoI: 5'-CTTACCTTCGAATGGGTGACAAAATTGCATTATGCTTTGAATTC-3' | This paper |  |
| Forward restriction primer for cloning FMR1's 3'UTR containing the 5' EcoRI site: 5'-TACTGAATTCAGGAGCAACAGCTCACAG-3' | This paper |  |
| Reverse restriction primer for cloning FMR1's 3'UTR containing the 3' XhoI site: 5'-ATACCTCGAGGCTTGATGGTTTGTGTTTTG-3' | This paper |  |
| Forward primer for amplifying the *ppk* 3’UTR: 5’-CACCTCGATGGTCTTAAAGGCCGAAAG-3’ | This paper |  |
| Reverse primer for amplifying the *ppk* 3’UTR: 5’-GCGAACACATTTTTTATTGTCGTG-3’ | This paper |  |
| Forward primer for amplifying the *chic* 3’UTR: 5’-CACCCCGCTTCCGTGGTAGAGAAACT-3’ | This paper |  |
| Reverse primer for amplifying the *chic* 3’UTR: 5’-TGACTTTGGGAACCGCGATA-3’ | This paper |  |

**REFERENCES:**

1. Zhang YQ, Bailey AM, Matthies HJ, Renden RB, Smith MA, Speese SD, et al. Drosophila fragile X-related gene regulates the MAP1B homolog Futsch to control synaptic structure and function. Cell. 2001;107(5):591-603. Epub 2001/12/06. doi: 10.1016/s0092-8674(01)00589-x. PubMed PMID: 11733059.
