# Supplementary material for: FXS causing missense mutations disrupt FMRP granule formation, dynamics, and function
Source: PLoS Genet. 2022 Feb 24;18(2):e1010084. doi: 10.1371/journal.pgen.1010084 (PMC8903291; doi:10.1371/journal.pgen.1010084)
Supplement: S3 Table — (DOCX) [file pgen.1010084.s006.docx]

**S3 Table: smFISH probes**

| REAGENT or RESOURCE | SOURCE | IDENTIFIER |
| --- | --- | --- |
| Oligonucleotides | | |
| camkii_1: 5’- GAAAAACGCGTACAGGCTGC-3’ Quasar 670 | LGC Biosearch Technologies | SS631302-01 |
| camkii_2: 5’- CCAACTCTTCTTTGATGTCG-3’ Quasar 670 | LGC Biosearch Technologies | SS631302-02 |
| camkii_3: 5’- GCAGCAAATTCAAAGCCAGT-3’ Quasar 670 | LGC Biosearch Technologies | SS631302-03 |
| camkii_4: 5’- CACTATGTTGGGATGGTGTA-3’ Quasar 670 | LGC Biosearch Technologies | SS631302-04 |
| camkii_5: 5’- CTCCTGTATACTGTCATGTA-3’ Quasar 670 | LGC Biosearch Technologies | SS631302-05 |
| camkii_6: 5’- AATGTGATGCATCAGCTTCT-3’ Quasar 670 | LGC Biosearch Technologies | SS631302-06 |
| camkii_7: 5’- CATTTTGGTGGCAGTGATTG-3’ Quasar 670 | LGC Biosearch Technologies | SS631302-07 |
| camkii_8: 5’- ATTCTCTGGTTTCAGATCTC-3’ Quasar 670 | LGC Biosearch Technologies | SS631302-08 |
| camkii_9: 5’- AGACCAAAGTCAGCGAGTTT-3’ Quasar 670 | LGC Biosearch Technologies | SS631302-09 |
| camkii_10: 5’- CTGATGATCGCCTTGAACTT-3’ Quasar 670 | LGC Biosearch Technologies | SS631302-10 |
| camkii_11: 5’- CTCCTTTTTCAATACCTCAG-3’ Quasar 670 | LGC Biosearch Technologies | SS631302-11 |
| camkii_12: 5’- AAGAATAACTCCACATGCCC-3’ Quasar 670 | LGC Biosearch Technologies | SS631302-12 |
| camkii_13: 5’- TGCTGATCTTCATCCCAAAA-3’ Quasar 670 | LGC Biosearch Technologies | SS631302-13 |
| camkii_14: 5’- ACGGATAATCATAAGCTCCC-3’ Quasar 670 | LGC Biosearch Technologies | SS631302-14 |
| camkii_15: 5’- TTTAGCTTCTGGAGTAACCG-3’ Quasar 670 | LGC Biosearch Technologies | SS631302-15 |
| camkii_16: 5’- GATGTTTTAAAGCCTCAGCT-3’ Quasar 670 | LGC Biosearch Technologies | SS631302-16 |
| camkii_17: 5’- CACACGTTCGCGTTGACAAA-3’ Quasar 670 | LGC Biosearch Technologies | SS631302-17 |
| camkii_18: 5’- CTTGAGACAGTCTACGGTTT-3’ Quasar 670 | LGC Biosearch Technologies | SS631302-18 |
| camkii_19: 5’- CGCCAACATTGTCGTAAGTA-3’ Quasar 670 | LGC Biosearch Technologies | SS631302-19 |
| camkii_20: 5’- GTTATCATACTTCTGCTCGA-3’ Quasar 670 | LGC Biosearch Technologies | SS631302-20 |
| camkii_21: 5’- GTTGATTCTTTGACCTGTGA-3’ Quasar 670 | LGC Biosearch Technologies | SS631302-21 |
| camkii_22: 5’- CGTCTTCAAGAGTAGTGCTA-3’ Quasar 670 | LGC Biosearch Technologies | SS631302-22 |
| camkii_23: 5’- GCCACTGTTAATTGCTTCAA-3’ Quasar 670 | LGC Biosearch Technologies | SS631302-23 |
| camkii_24: 5’- CAAAGGCAGTTAGATGCGGA-3’ Quasar 670 | LGC Biosearch Technologies | SS631302-24 |
| camkii_25: 5’- ATTCCTTCTACAAGGTTACC-3’ Quasar 670 | LGC Biosearch Technologies | SS631302-25 |
| camkii_26: 5’- GCTTTGCAGTTTTTACCAAG-3’ Quasar 670 | LGC Biosearch Technologies | SS631302-26 |
| camkii_27: 5’- CTTCACCAAGTAAGTGCACA-3’ Quasar 670 | LGC Biosearch Technologies | SS631302-27 |
| camkii_28: 5’- GTCTCACATAGGCAATGCAA-3’ Quasar 670 | LGC Biosearch Technologies | SS631302-28 |
| camkii_29: 5’- CATTCTGCCATTTGTTATCG-3’ Quasar 670 | LGC Biosearch Technologies | SS631302-29 |
| camkii_30: 5’- CTTATTTTGGCAGATGCACT-3’ Quasar 670 | LGC Biosearch Technologies | SS631302-30 |
| chic-570-1: 5’- CCGCAACACCGACGATTT-3’ Quasar 570 | LGC Biosearch Technologies | SS630980-01 |
| chic-570-2: 5’- CGGGGGTCACACGAAATT-3’ Quasar 570 | LGC Biosearch Technologies | SS630980-02 |
| chic-570-3: 5’- CGAGTCGCACTTTCGTTT-3’ Quasar 570 | LGC Biosearch Technologies | SS630980-03 |
| chic-570-4: 5’- TGTTGCTTTACCGCACGG-3’ Quasar 570 | LGC Biosearch Technologies | SS630980-04 |
| chic-570-5: 5’- GATCTGGATATGGATCGC-3’ Quasar 570 | LGC Biosearch Technologies | SS630980-05 |
| chic-570-6: 5’- GTCGTGGGTGCGGATTAA-3’ Quasar 570 | LGC Biosearch Technologies | SS630980-06 |
| chic-570-7: 5’- GCTCATGGTGCTTTGTTT-3’ Quasar 570 | LGC Biosearch Technologies | SS630980-07 |
| chic-570-8: 5’- GTCCACATAATCTTGCCA-3’ Quasar 570 | LGC Biosearch Technologies | SS630980-08 |
| chic-570-9: 5’- CGAGGCCAGGAGTTGGTT-3’ Quasar 570 | LGC Biosearch Technologies | SS630980-09 |
| chic-570-10: 5’- GATGCACGCCTTGGTCAC-3’ Quasar 570 | LGC Biosearch Technologies | SS630980-10 |
| chic-570-11: 5’- CCAAATGTTGCCGTCGTG-3’ Quasar 570 | LGC Biosearch Technologies | SS630980-11 |
| chic-570-12: 5’- TTTCCTCTGCTACACACA-3’ Quasar 570 | LGC Biosearch Technologies | SS630980-12 |
| chic-570-13: 5’- GCATTTTTACTCGATCCA-3’ Quasar 570 | LGC Biosearch Technologies | SS630980-13 |
| chic-570-14: 5’- CCGCTGATCAGTTTGGAG-3’ Quasar 570 | LGC Biosearch Technologies | SS630980-14 |
| chic-570-15: 5’- CCGTCCTGCTGGTCAAAG-3’ Quasar 570 | LGC Biosearch Technologies | SS630980-15 |
| chic-570-16: 5’- AGTGTCACGCCGTTGCTG-3’ Quasar 570 | LGC Biosearch Technologies | SS630980-16 |
| chic-570-17: 5’- TAAATGTACCGCTGGCCG-3’ Quasar 570 | LGC Biosearch Technologies | SS630980-17 |
| chic-570-18: 5’- CGGTCTGTGCCGGAAAGG-3’ Quasar 570 | LGC Biosearch Technologies | SS630980-18 |
| chic-570-19: 5’- GTCTTCATGCAGTGCACT-3’ Quasar 570 | LGC Biosearch Technologies | SS630980-19 |
| chic-570-20: 5’- ACGATCACGGCTTGTGTT-3’ Quasar 570 | LGC Biosearch Technologies | SS630980-20 |
| chic-570-21: 5’- GGGATCCTCGTAGATGGA-3’ Quasar 570 | LGC Biosearch Technologies | SS630980-21 |
| chic-570-22: 5’- TCTCTACCACGGAAGCGG-3’ Quasar 570 | LGC Biosearch Technologies | SS630980-22 |
| chic-570-23: 5’- CTATTCTCCTAGTACCCG-3’ Quasar 570 | LGC Biosearch Technologies | SS630980-23 |
| chic-570-24: 5’- TCATTTACGGTTCGCTCT-3’ Quasar 570 | LGC Biosearch Technologies | SS630980-24 |
| chic-570-25: 5’- GTTGGTTTTTCTTTTCCC-3’ Quasar 570 | LGC Biosearch Technologies | SS630980-25 |
| chic-570-26: 5’- TTTCCTCTGCTACACACA-3’ Quasar 570 | LGC Biosearch Technologies | SS630980-26 |
| chic-570-27: 5’- GCATTTTTACTCGATCCA-3’ Quasar 570 | LGC Biosearch Technologies | SS630980-27 |
